# Long- COVID and general health status in hospitalized COVID-19 survivors

**DOI:** 10.1038/s41598-023-35413-z

**Published:** 2023-05-19

**Authors:** Zahra Mohtasham-Amiri, Fatemeh Keihanian, Enayatollah Homaie Rad, Reza Jafari Shakib, Leila Kanafi Vahed, Leila Kouchakinejad–Eramsadati, Seyed Mahmoud Rezvani, Rastin Nikkar

**Affiliations:** 1 Preventive and Community Medicine Department, School of Medicine, Guilan University of Medical Sciences, Rasht, Iran; 2 Guilan Road Trauma Research Center, Guilan University of Medical Sciences, Rasht, Iran; 3 Social Determinants of Health Research Center, Guilan University of Medical Sciences, Rasht, Iran; 4 Guilan Road Trauma Research Center, Guilan University of Medical Sciences, Rasht, Iran; 5 Department of Immunology, School of Medicine, Guilan University of Medical Sciences, Rasht, Iran; 6 Anesthesiology Department, School of Medicine, Guilan University of Medical Sciences, Rasht, Iran

**Keywords:** Diseases, Medical research

## Abstract

Despite advances in clinical research, the long-term effects of COVID-19 on patients are not clear. Many studies revealed persistent long-term signs and symptoms. In a survey study, 259 hospitalized confirmed COVID-19 patients between 18 and 59 years were interviewed. Demographic characteristics and complaints were studied through telephone interviews. Any patient-reported symptoms that continued or developed from 4 weeks up to 12 weeks after the onset of the disease were recorded only if they did not exist prior to infection. The 12-Item General Health Questionnaire was used for screening and assessing mental symptoms and psychosocial well-being. The mean age of participants was 43.8 ± 9.9 years. About 37% had at least one underlying disease. 92.5% showed ongoing symptoms that the most prevalent complications were hair loss (61.4%), fatigue (54.1%), shortness of breath (40.2%), altered smell (34.4%), and aggression (34.4%), respectively. In terms of factors affecting patients' complaints, there were significant differences between age, sex, and underlying disease with long-remaining complications. This study shows a high rate of long COVID-19 conditions that should be considered by physicians, policymakers, and managers.

## Introduction

Globally, as of May 2022, more than 500 million confirmed cases of COVID-19, and over 6 million deaths have been reported to the World Health Organization^1^.

Most people infected with COVID-19 recover spontaneously and do not need any treatment or hospitalization. However, about 10% to 15% of patients develop severe pneumonia, and about 5% of critically ill patients would progress to acute respiratory distress syndrome (ARDS) and multiorgan failure, requiring intensive care units^2,3^. The mortality rate varies depending on the underlying disease, age, and health care access^3^.

There has not been a consensus on describing the condition that COVID-19 symptoms persist after the initial phase^2^. “Long covid” is a patient-coined term indicating the state of persisting symptoms of COVID-19 after recovery from acute illness for several weeks and months^4^. The National Institute for Health and Care Excellence defined long COVID as signs and symptoms developed during or following a disease consistent with COVID-19 and which continue for more than four weeks but cannot be explained by alternative diagnoses^5^. This term consists of two phases: ongoing symptomatic COVID (4–12 weeks), and post-COVID syndrome (more than 12 weeks)^6^. The World Health Organization (WHO) has provided a new case definition as the post-COVID-19 condition which is a history of probable or confirmed SARS CoV-2 infection, usually 3 months from the onset of COVID-19 with symptoms and that last for at least 2 months and cannot be explained by an alternative diagnosis. Symptoms can be persistent, new-onset, fluctuating, or relapsing over time^7^. Regardless of the severity of the primary infection or prior health status, any patient with COVID-19 may experience long-term COVID-19 complications^8^. Although persistent symptoms are often correlated with the severity of symptoms in the acute phase of the infection^9,10^, they can also occur following a mild illness^11,12^. Researchers hypothesized that the long-term complications of COVID-19 are related to the ability of SARS-CoV-2 to trigger an extensive inflammatory response^12^. Studies have shown that 31% to 69% of COVID-19 patients experience several symptoms beyond four or more weeks^13^. The commonly reported symptoms are fatigue, shortness of breath, myalgia, cough, headache, chest pain, hair loss, brain fog, smell and taste disorders, sleep difficulties, and depression^2,12,14^. These symptoms negatively impact daily activities, cognitive functions, and the patient’s quality of life^15^. Long COVID has health, work, social, and economic consequences. A UK cohort study on COVID-19 patients following hospitalization revealed that one in five patients hospitalized with COVID-19 did not return to work five months later. A similar proportion had changed their job because of the health issues^16^. Iran is one of the most affected countries by the COVID-19 pandemic, with more than 140,000 deaths, at the time of writing the article^17,18^. In this study, we aimed to assess the ongoing complications of hospitalized COVID-19 patients and their general health status in northern Iran.

## Methods

In a survey study, COVID-19 survivors who admitted to the Razi tertiary referral hospital of Guilan, northern Iran, during the Delta surge from July to September 2021 were selected. The inclusion criteria were: (1) patients aged between 18 and 59 years, (2) confirmed SARS-CoV-2 infection by a positive reverse-transcriptase polymerase chain reaction (RT-PCR) assay, (3) moderate to severe COVID-19 infection, and (4) passing at least three months after the initial infection of participants. Patients who were admitted to the intensive care unit, those who died in hospital, or had life-threatening underlying diseases such as advanced disseminated malignancy were removed from the study. Finally, of all the hospital information system (HIS) records, 319 patients were selected by a simple random sampling method as below formula:

$${\text{n}} = {\text{NZ}}^{2}_{1} - \upalpha /_{2} {\text{p}}\left( {1 - {\text{p}}} \right)/{\text{d}}2\left( {{\text{N}} - 1} \right) + {\text{ Z}}^{2}_{1} - \upalpha /2{\text{p}}\left( {1 - {\text{p}}} \right)$$

$$\upalpha = 0.0{5}\;\;\;{\text{p}} = 0.{35},\;\;\;{\text{d}} = 0.0{35},\;\;\;{\text{N}} = {57}0,\;\;\;{\text{n}} = { 319}$$

From the total list, we selected the patinets using the random function of Excell software. Those subjects who refused participating in the survey was replaced by another one. Demographic characteristics and medical history were retrieved from the hospital data bank. A comprehensive checklist containing 30 known complications of long COVID-19 was designed using review articles and interviews with health professionals, infection disease specialists, dermatologists, cardiologists, and neurologists.

The checklist encompasses neurological symptoms (headache, dizziness, memory impairment, smell or taste disorder, paresthesia, tinnitus, hypoacusis, visual disturbances), autonomic symptoms (chest pain, palpitations), gastrointestinal symptoms (diarrhea, abdominal pain, gastroesophageal reflux, and stomachache), respiratory symptoms (shortness of breath, cough), musculoskeletal symptoms (fatigue, myalgia, and arthralgia), psychological symptoms (aggression, anxiety, depression, insomnia, nightmare), and dermatological symptoms (hair loss, skin rashes), and other manifestations (sexual dysfunction, menstrual disorder, leg swelling).

Participants were interviewed using a semi-structured interview method. A single trained physician conducted telephone interviews with COVID-19 survivors based on the admission date documented in their medical records. If patients did not respond to the first call, it was repeated after 3 days. If failed, the call was made a week later (up to three times). Individuals who did not respond after three unsuccessful attempts, those who declined to participate in the study, and patients who died post-discharge or could not answer the questions were excluded during the study.

Symptoms were documented using the designed checklist. Any patient-reported symptoms that continued or developed from 4 weeks up to 12 weeks after the onset of the COVID-19 were recorded only if they did not exist prior to infection. Furthermore, patients were asked to report any other complaints, exacerbations of pre-existing underlying diseases, or newly diagnosed diseases after acute COVID-19 infection. In addition, the 12-item General Health Questionnaire (GHQ) was conducted to screen for mental health, mental distress, and social dysfunction^19^.

Participation was voluntary, and informed consent was obtained from all participants after explaining the aim of the research. The study was approved ethically by the ethics committee of Deputy of Research, Guilan University of Medical Sciences (IR.GUMS.REC.1400.052). All methods were carried out in accordance with the ethical standards as laid down in the 1964 Declaration of Helsinki and its later amendments or comparable ethical standards. Data analyses were done using Excel and STATA SE software v13.1.

Mean and standard deviation were used for presenting continuous variables. Binary and categorical variables were presented as counts and percentages. Non-parametric tests such as the χ^2^ test were used to identify differences in proportions across multiple categories. All tests were two-tailed, and p values of less than 0.05 were considered statistically significant.

## Results

Telephone interviews were conducted with 319 patients from December 2021 to February 2022. Two patients died after discharge. Out of 317 patients, follow-up was accomplished in 259 COVID-19 survivors. The survey response rate was 81.7%.

Based on the follow-up results, the majority of the participants were female (58.3%). Most of the patients had a high school diploma, 73 (27.9%) had academic degrees, and 13 (5%) were illiterate. The mean age of participants was 43.8 ± 9.9 years (ranging from 18 to 59 years), and the mean length of hospital stay was 4.4 ± 4 days (1–48 days). Overall, 22 (8.56%) were vaccinated with at least a single dose of COVID-19 vaccine on admission. Twenty-five patients (9.65%) were smokers. Ninty (34.7%) patients were self-employed, 14 (5.4%) were retired, and 112 (43.2%) were housewives. Of all 259 participants, 163 (63%) had no background diseases, and 96 (37%) had at least one chronic disease. The most common comorbidities were diabetes mellitus in 38.1%, hypertension in 32%, hypothyroidism in 13.4%, dyslipidemia in 10%, thalassemia minor in 6.1%, and coronary artery diseases in 3%. Participants with a history of underlying disease reported 21% more symptoms than the others. In 77% of the patients, at least one family member living under the same roof was infected simultaneously (Table 1).

**Table 1:** Demographic and clinical characteristics of participants.

Variable	Number of subjects (%)
Education status	
Illiterate or < 5 grade	41 (15.8)
5–9 grade	55 (21.2)
9–12 grade	90 (34.7)
> 12 grade	69 (26.7)
Missing data	4 (1.6)
Sex	
Female	151 (58.3)
Male	108 (41.7)
Age group (years)	
< 30	26 (10.1)
30–39	54 (20.8)
40–49	94 (36.3)
50–59	85 (32.8)
Marital status	
Single	5 (1.9)
Married	254 (98.1)
Job status	
Self-employed	90 (34.7)
Retired	14 (5.4)
Unemployed	4 (1.5)
Housewife	112 (43.2)
Student	7 (2.7)
Employee	12 (4.5)
Manual worker	13 (5)
Underlying diseases	
No	163 (63)
Yes	96 (37)
Vaccination status	
Not vaccinated	235 (91.4)
Vaccinated (single dose)	22 (8.6)
Smoking status	
No	234 (90.4)
Yes	25 (9.6)

**Figure 1:**
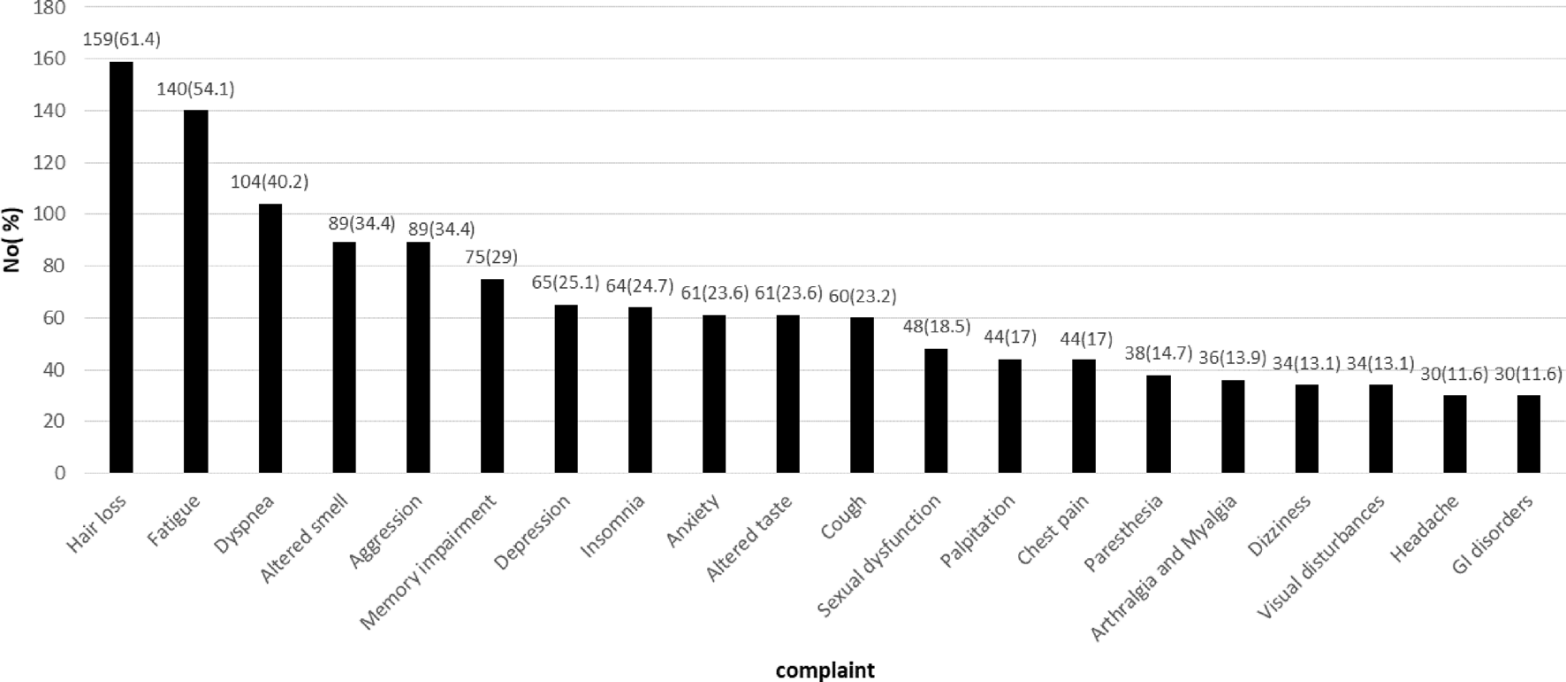
The prevalence of the most common symptoms in COVID-19 patients after initial infection.

Of all participants, 7.5% had no complaints and the majority of them (40.2%) had more than 5 complaints. The most prevalent complications were hair loss (61.4%), fatigue (54.1%), shortness of breath (40.2%), altered smell (34.4%), aggression (34.4%), memory impairment (29%), and self-perceive depression (25.1%), respectively. Figure 1 shows the prevalence of the most common symptoms after infection.

Of all women who were not menopaused (n = 99), 28 (28.3%) complained of menstrual cycle irregularities, and 13 (13.1%) suffered from AUB (abnormal uterine bleeding). Dysgeusia, parosmia, and phantosmia were seen in 29 (11.2%), 23 (8.9%), and 23 (8.9%), respectively. Gastrointestinal (GI) problems consisted of diarrhea, abdominal pain, gastroesophageal reflux, and stomachache. Four people complained of newly diagnosed diabetes mellitus after COVID-19, and four cases also complained of worsening their pre-existing diabetes and insulin dependency after hospital discharge (Fig. 1).

The results of GHQ-12 showed that the mean score was 1.53 ± 2.66 in the range of 0 and 12. Furthermore, 31 patients faced depression (11.11%) based on the GHQ-12 results. In terms of general health status, according to the cut-off point of 3.5 in the study, 52 people (20.2%) were classified as having mental health problems. According to the t-test, there was no statistical difference between men and women.

In terms of factors affecting patients' complaints, Poisson regression analysis was used to show the effective factors of complaint after COVID-19. As shown in Table 2, the Variance Inflation factor was lower than 10, which means that there was not a collinearity effect between symptoms. there were no statistically significant differences between occupation, length of hospital stay and the remaining complaints. However, there were significant differences between age, level of education, sex, and background disease with long complications (Age coefficient was 0.008, 95% CI [0.002 to 0.013]; male sex coefficient was − 0.251, 95% CI [− 0.402 to − 0.100]; background disease coefficient was 0.147, 95% CI 0.036 to 0.259] (Table 2).

**Table 2:** Poisson regression analysis to show the effective factors of complaint counts after COVID-19.

Complaint total	Coefficient	standard error	P-value	95% lower limit	95% upper limit
Age	0.008	0.003	0.011	0.002	0.013
Sex (male)	− 0.251	0.077	0.001	− 0.402	− 0.100
Married	0.541	0.272	0.046	0.009	1.074
Educational level (illiterate-base)					
5–9 grade	− 0.426	0.136	0.002	− 0.693	− 0.160
9–12 grade	− 0.110	0.116	0.344	− 0.338	0.118
> 12 grade	0.022	0.114	0.849	− 0.201	0.245
Background disease (No)	0.147	0.057	0.010	0.036	0.259
Job status (unemployed-base)					
Employed	− 0.256	0.225	0.256	− 0.697	0.185
Out of labor participation	− 0.111	0.226	0.623	− 0.555	0.332
Constant variable	1.172	0.340	0.001	0.505	1.839
Variance Inflation factor (VIF test) = 5.87					

LR Chi^2^ = 109.69.

Pseudo R^2^ = 0.3269.

According to the type of remaining complaints, fatigue (ρ = 0.022), headache (ρ = 0.011), dizziness (ρ = 0.025), visual disturbance (ρ = 0.025), hair loss (ρ = 0.0001), and anxiety (ρ = 0.003) were significantly more common in women compared with men (χ^2^ test).

## Discussion

In the present study, we assessed the long-term COVID-19-related symptoms in hospitalized patients in the non-intensive COVID-19 wards of a tertiary referral center, 3 months after infection, during the fifth pandemic wave in northern Iran. The results showed that 7.5% of participants were symptom-free and the only complaint in 23.2% of the cases was the hair loss. In a study performed on hospitalized COVID-19 survivors, 89.0% of patients had at least one symptom 3 months later^20^. A new meta-analysis reported that 80% (95% CI 65–92) of the patients with COVID-19 experienced at least one or more long-term symptoms^21^.

Arnold DT, in a prospective follow-up study in the UK, demonstrated that about 74% of COVID-19 patients appeared symptomatic 3 months after hospitalization^22^. In some reports assessing COVID-19 patients at 60 days post-discharge, the proportion of patients with at least one persistent COVID-19 symptom ranged from 66 to 87%^23–25^. A longitudinal study in Wuhan, China illustrated that 49.6% of the hospitalized patients continue to suffer from at least one symptom including physical deterioration, fatigue, and myalgia, after 3 months of discharge^26^.

Reports have shown that 1 in 5 patients, regardless of the severity of the initial infection, may complain of COVID-related persistent symptoms for 5 weeks or more, while 1 in 10 may have symptoms lasting 12 weeks or more^2^.

One study reported persistent symptoms after 60 days on outpatients as lower rate 27%^27^. The causes of considerable differences in long COVID proportion among studies seem to be the difference in the type of participants (inpatient or outpatient), follow-up time from the acute episode of infection, the severity of disease, geographical area, power of tests to confirm the COVID-19 diagnosis, and the inability to track and follow up all patients.

The five commonest long COVID complaints in our study were hair loss, fatigue, shortness of breath, altered smell, and aggression. Our findings were consistent with previous studies that the most prevalent symptoms were fatigue and dyspnea, ranging from 35 to 60% according to the follow-up time^2,22,24,28,29^.

In a symptom cluster analysis comparing long COVID symptoms 30 days and 90 days after discharge, alopecia was the main complaint at 90 days^23^. In another study, 46.1% of hospitalized COVID survivors at 3 months experienced hair loss^20^. The hair shedding which occurs 2 to 3 months following a stressful event, including severe infective episodes, is known as Telogen effluvium (TE)^30^. Since COVID-19 is a febrile and infectious disease with high emotional and physiological stress, TE would be an expected manifestation. The medications administered during the acute phase can also play a role in the development of TE^31^. In our study, hair loss has highly affected women. Women are more attentive to hair loss because of its effects on beauty and psychological consequences. Additionally, the long hair of women is more easily identified during hair loss. COVID-related alopecia has been probably under-reported among men^30,32^.

In the majority of previous research, fatigue appeared as the most common prevalent symptom after recovery from acute COVID-19 infection^9,20,21,33^.

Shortness of breath was the most prevalent respiratory symptom experienced by our patients. Other studies have reported the prevalence of dyspnea ranging from 5 to 81%, 1 to 12 months after hospitalization^34^.

Among neurological symptoms, olfactory dysfunction was the most commonly reported complaint. Smell alteration is a frequent early symptom of COVID-19 infection, which is attributed to neuroinflammation of the olfactory bulb^35^. It usually subsides over a period of 2–3 weeks, however, it persists in some patients beyond a month due to prolonged healing^36^. In a systematic review, the most neurological symptoms were headache, insomnia, and loss of smell or taste^28^.

Another prominent complaint asserted by about one-third of our patients was aggression. However, in other studies, depression was frequently reported as the main psychiatric disorder^37^. In another study in Mexico City, among mood disorders, sadness, desire to cry, anguish, anger, and anhedonia were the most common symptoms reported^23^.

In the present study, there was a significant correlation between remained complaints and sex, and it was higher in women. This finding is in agreement with the previous studies^7,27,38,39^. It is attributable to the fact that women pay more attention to their health, and are more expressive.

Similar to the preliminary studies, we found that underlying comorbidities were significantly associated with the presence of long-term symptoms, and those with pre-existing diseases reported more complaints^14,15^.

There was a association between age and remained symptoms in the current study in consist of the Logue study^14^, that long-term complications were reported more frequently in the higher age groups.

Most infected patients with COVID-19 are asymptomatic or have mild symptoms. Therefore, measuring the exact prevalence of long COVID complications is not possible. The present study was performed only on hospitalized patients who had moderate to severe conditions and were expected to have a higher percentage of complications and complaints.

The current study did not assess the quality of life of patients. Their general health status was evaluated 6 months after COVID-19 infection, and the majority of the participants were in a good condition.

Our study had several limitations. It was a symptom-oriented assessment. We did not use objective evaluation, and the severity of reported symptoms was not assessed. However, symptoms were documented by a physician, and only reliable and significant symptoms were recorded after comprehensive history-taking. The relatively small sample size of the study may leave some associations undetected. Single-center design, the age limits, recall bias, and the exclusion of ICU-admitted patients and those with malignant comorbidities are other limitations. Moreover, this study was performed only on hospitalized patients and did not include outpatients. More extensive studies are required in order to better understand the epidemiology and clinical course of the long COVID condition.

## Data Availability

The data that support the findings of this study are available from the corresponding author upon reasonable request.
